# Sample Aging Profoundly Reduces Monocyte Responses in Human Whole Blood Cultures

**DOI:** 10.1155/2018/8901485

**Published:** 2018-06-05

**Authors:** H. W. Grievink, M. Moerland

**Affiliations:** Centre for Human Drug Research, Zernikedreef 8, 2333 CL Leiden, Netherlands

## Abstract

Human whole blood cultures are widely used for the investigation of physiological pathways and drug effects *in vitro*. Detailed information on the effect of “sample aging” (the time span between blood collection and experimental start) on the experimental outcome is not readily available in the public domain. We studied the effect of sample aging on the ability of immune cells to respond to cell-specific immune triggers (LPS, PMA/ionomycin, and SEB). Sample aging at room temperature profoundly inhibited the LPS-induced monocytic cytokine release in minimally diluted whole blood cultures. The reduction ranged from 20–50% after 30 minutes to 80–100% after 10 hours and differed between cytokines (IL-1*β*, IL-2, IL-6, IFN*γ*, and TNF*α*). Sample storage at 4°C or 37°C even worsened this. PMA/ionomycin- and SEB-induced cytokine release, both mainly T-cell-driven, were also reduced by sample aging but to a lesser extent (20–50% after 24 hours). Intracellular cytokine staining revealed that the number of LPS-responding cells was not impacted by sample aging and reduced LPS responsivity could also not be explained by apoptosis or downregulated TLR4 expression. Thus, we speculate that sample aging induces an inhibitory pathway downstream from TLR4 in monocytes. These results underline the importance of quick sample handling when investigating innate immune responses in whole blood, especially for monocyte responses.

## 1. Introduction

Primary human immune cells are widely used for the investigation of physiological pathways and drug effects *in vitro*. Primary cells are often used for research on chronic diseases and for testing new therapies for a wide range of diseases. Such experiments are frequently performed on isolated leukocytes, mostly peripheral blood mononuclear cells (PBMCs). Cryopreserved PBMCs can be analyzed batch-wise, so that samples collected at multiple clinical sites can be analyzed at one central laboratory. Alternatively, *in vitro* experiments can be conducted on whole blood samples, with the obvious benefit that this better resembles *in vivo* conditions [1]. Sample handling is minimal for *in vitro* experiments on whole blood samples: the only variable to control is the time span between blood collection and the start of the experiment. Although most publicly available literature on whole blood-based experiments does not specify this time span, it is evident that the “sample age” may vary substantially between and within experiments, depending on the clinical unit, the laboratory, and the donor population involved. Limited information is available on the effect of the sample age on the outcome of whole blood-based experiments. This is remarkable since a direct relationship between sample age and cell functionality (i.e., cell viability and cell responsiveness to immune triggers) could be expected. In PBMC cultures, apoptosis occurs spontaneously after prolonged sample storage [2, 3]. In whole blood samples, blood settling causes red blood cell and platelet aggregation and alters cell function [4]. Another potential problem in whole blood cultures is the short lifespan of neutrophils [5]. These cells survive for less than 24 hours in the bloodstream and are prone to undergo apoptosis under suboptimal environmental conditions. Apoptotic neutrophils may have secondary effects on other leukocyte subsets in a whole blood-based experiment. To overcome these problems, efforts have been made to preserve whole blood samples for longer periods of time. For example, cryopreservation of the whole blood has been shown to be feasible for experiments assessing Epstein-Barr virus (EBV) transformation, lymphocyte proliferation, and DNA extraction [6–8]. Freezing of fixed whole blood may also be appropriate for immunophenotyping [9]. However, granulocytes do not stay viable during the freezing process impacting the responses of other cell types [10, 11]. The addition of phytohaemagglutinin (PHA) during sample storage avoided the apoptosis of lymphocytes [2], and the addition of the polymer Ficoll to the whole blood prevented settling of red blood cells, limiting interference of aggregating red blood cells [4]. However, in addition, such chemicals may induce undesired cellular activation: PHA, for example, is known to stimulate T-cell proliferation [12]. An experimental setup without the addition of cell-preserving chemicals is preferred when studying the natural behavior and effect of blood cells.

A limited number of studies describe the effects of whole blood storage on cellular responses. Unfortunately, these studies only focus on the effects on a specific cell population or cellular response, with contradictory results. The temperature at which the blood is stored may affect the functionality of monocytes, with low temperature storage (4°C for 24 hours) preferred over room temperature [13]. Another study demonstrated that when stored at room temperature, the number of cytokine-producing monocytes remains relatively stable, whereas interferon (IFN)*γ*- and IL-2-producing T-cells declined during storage [14].

We aimed to provide a comprehensive overview of the effect of sample aging on cell viability and stress and cellular reactivity to exogenous immune triggers. We investigated cell responsiveness by quantification of secreted cytokines, and we looked at the percentage of responsive cells for particular cell subsets (T-cells and monocytes) by means of the flow cytometric detection of intracellular cytokines. To obtain insight into cell-specific or pathway-specific effects of sample aging, we used immune triggers activating different immune cell subsets. T-cells were stimulated by simultaneous incubation with phorbol 12-myristate 13-acetate (PMA) and ionomycin and by staphylococcal enterotoxin B (SEB). PMA plus ionomycin induces a general T-cell activation via protein kinase C (PKC) and nuclear factor of activated T-cell (NFAT) signaling. Superantigen SEB activates up to 20% of all T-cells via the T-cell receptor *β* chain [15]. Monocyte activation was induced by lipopolysaccharide (LPS), a Toll-like receptor (TLR) 4 ligand.

## 2. Materials and Methods

### 2.1. Blood Collection

Blood was collected from healthy volunteers by venipuncture into sodium heparin-coated vacutainers or cell preparation tubes (CPT) containing sodium heparin (Becton Dickinson, NJ, USA) after written informed consent was obtained in accordance with Good Clinical Practice guidelines and the Declaration of Helsinki.

### 2.2. Whole Blood Incubations

Whole blood was simulated for 3 or 24 hours with LPS (2 ng/mL), SEB (100 ng/mL), or PMA/ionomycin (150 ng/mL and 7.5 *μ*g/mL, resp.). All reagents were obtained from Sigma-Aldrich (Deisenhofen, Germany). For intracellular staining, brefeldin A (Thermo Fisher Scientific) was added to the cultures. Cultures were incubated at 37°C and 5% CO_2_.

### 2.3. PBMC Isolation and Incubations

PBMCs were collected from CPT samples and washed twice with PBS. PBMCs were counted using the MACSQuant 10 analyzer and resuspended at 1 × 10^6^ cells/mL in RPMI1640 supplemented with 10% FBS. PBMCs were stimulated with 2 ng/mL LPS for 24 hours.

### 2.4. Flow Cytometry

Flow cytometric staining of leukocyte subsets in whole blood cultures was done after red blood cell lysis with RBC lysis buffer (Thermo Fisher Scientific). Leukocytes were incubated with TLR4-APC (clone HTA125, Thermo Fisher Scientific), Annexin V-FITC (Miltenyi Biotec), CD14-VioBlue (clone REA599), and/or CD3-VioGreen (clone REA613) for 30 minutes at 4°C. For intracellular staining, cells are fixed (IC fixation buffer, Thermo Fisher Scientific) and permeabilized (permeabilization buffer, Thermo Fisher Scientific). Then IL-2-PE (clone N7.48 A), TNF*α*-PE (clone REA656), IFN*γ*-PE (clone REA600), or IL-6-PE (clone MQ2-13A5) antibodies were added in the presence of Fc Receptor blocking reagent (all from Miltenyi Biotec, Bergisch-Gladbach, Germany, unless indicated otherwise) and incubated at 4°C for 30 minutes. Samples were measured with a MACSQuant 10 analyzer (Miltenyi Biotec). Unstimulated and fluorescence minus one controls for nonlineage markers were used for gate setting.

### 2.5. Mitochondrial Function

Mitochondrial membrane potential (MMP) was assessed in whole blood cultures after red blood cell lysis with RBC lysis buffer. Leukocytes were incubated with 0.5 *μ*M JC-1 (Mitoprobe kit, Thermo Fisher Scientific) for 15 minutes. CCCP (10 *μ*M) was used as a positive control. MMP was assessed in monocytes and T-cells with a MACSQuant 10 analyzer (Miltenyi Biotec). Mitochondrial function was expressed as the mitochondrial membrane potential, calculated as follows [16]:

(1) $$\begin{array}{c} \Delta \psi m=\frac{\left(FL1:FL2\right)}{\left(FL1\_CCCP:FL2\_CCCP\right)}*100\%. \end{array}$$

### 2.6. Cytokine Measurements

IFN*γ*, TNF*α*, IL-1*β*, and IL-6 from whole blood culture supernatants were measured using the V-plex inflammatory panel-I kit from Meso Scale Discovery (Rockville, MD, USA). IL-2 and IL-10 were measured by ELISA (Thermo Fisher Scientific).

## 3. Results

### 3.1. Sample Aging Results in a Rapid Decline of LPS-Driven Responses, Whereas PMA- and SEB-Driven Are Less Affected

**Figure 1:**
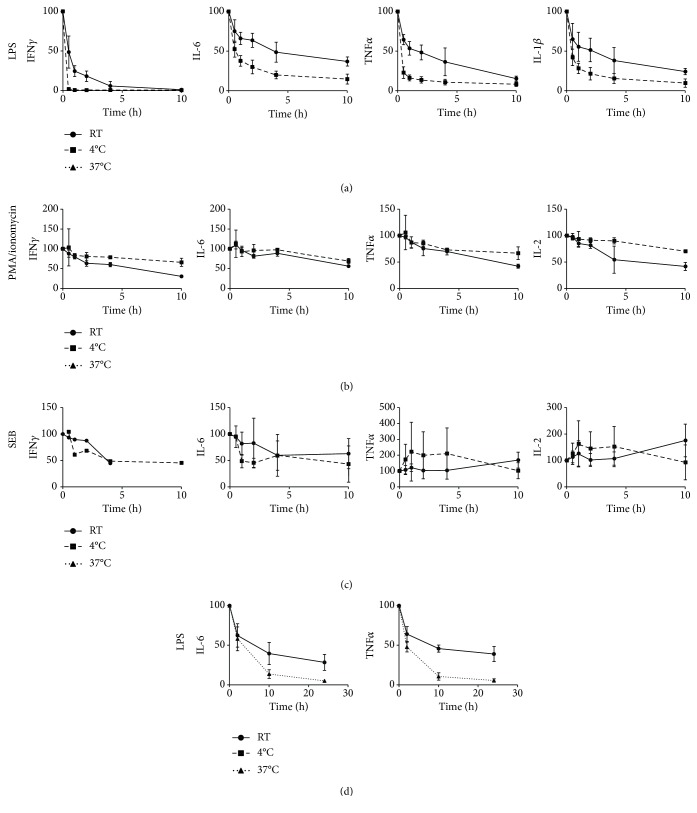
Cytokine release in whole blood culture supernatants (as % of the response at *t* = 0, average plus SD). The whole blood of 3 donors was stimulated for 24 hours with LPS (a), PMA/ionomycin (b), or SEB (c). The whole blood of 5 donors was stimulated for 24 hours with LPS (d). The *x*-axis indicates the sample age (time span between blood collection and start incubation). The blood was stored at room temperature (continuous line), 4°C (dashed line (a–c)), or 37°C (dashed line (d)).

Whole blood was collected and stored at room temperature or at 4°C until incubation experiments were started (immediately after blood collection and 0.5, 1, 2, 4, and 10 hours after blood collection). Whole blood cultures were stimulated with LPS, SEB, and PMA/ionomycin. Sample aging strongly reduced LPS-induced IFN*γ*, IL-1*β*, IL-6, and TNF*α* release (Figure 1(a)). A delay in the start of the incubation of only 0.5 hour already resulted in a loss of 30–50% of the cytokine response when stored at room temperature. When the samples were stored at 4°C, the decrease in cell responses was even more drastic, ranging from 50% for IL-6 to 98% for IFN*γ*. Interestingly, when the whole blood was stimulated with PMA/ionomycin, again, an effect of sample aging on cytokine release was observed, but this effect was much less profound (Figure 1(b)). After 0.5 hour, hardly any effect of sample aging was observed. Maximal aging of 10 hours resulted in a loss of 25–60% of the cytokine response (IFN*γ*, IL-6, and TNF*α*). In contrast to LPS responses, sample storage at 4°C better preserved PMA/ionomycin-induced cytokine release compared to storage at room temperature. SEB responses were highly variable between subjects, but no strong indications were found for a time-dependent reduction in cell responses (Figure 1(c)). Cytokine release in unstimulated samples remained low (<50 pg/mL) for all sample ages investigated (data not shown). Sample aging did not induce the release of the anti-inflammatory cytokine IL-10 (all responses < 25 pg/mL, data not shown).

Given the large temperature-dependent effect on LPS-driven responses, sample storages at 37°C and at room temperature were compared in a separate experiment (Figure 1(d)). The whole blood was stimulated with LPS immediately after blood collection and after 2, 10, and 24 hours of storage. Sample storage at 37°C resulted in an even more rapid decrease in IL-6 and TNF*α* production compared to room temperature.

### 3.2. Sample Aging Does Not Affect Cell Viability, Mitochondrial Function, or TLR4 Expression

**Figure 2:**
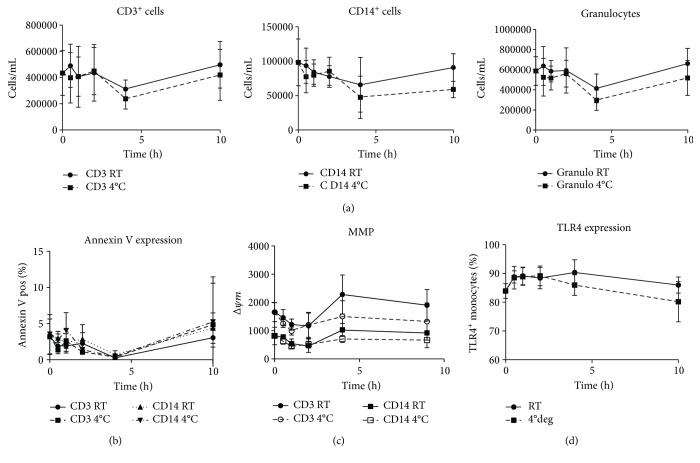
Cell viability (cell counts, annexin V, and MMP) and TLR4 expression in whole blood cultures (average plus SD). In whole blood cultures of 6 donors, absolute cell counts (T-cell, monocyte and granulocyte numbers (a)), apoptosis (annexin V-positive T-cells and monocytes (b)), mitochondrial function (MMP for T-cells and monocytes (c)), and TLR4 expression (TLR4-positive monocytes (d)) were quantified. The *x*-axis indicates the sample age (time span between blood collection and start incubation). The blood was stored at room temperature (continuous lines) or 4°C (dashed lines).

Since LPS stimulation results in a mainly monocyte-driven response and SEB and PMA/ionomycin are mainly T-cell stimuli, we hypothesized that monocytes are more prone to undergo cell death due to sample aging. However, absolute counts of CD3^+^ T-cells, CD14^+^ monocytes, and granulocytes (as gated in FSC/SSC scatter plot) did not decrease with prolonged sample storage (Figure 2(a)). Also no increase in the number of dead cells was observed, as measured by propidium iodide staining (data not shown). To check whether sample aging rendered the cells apoptotic, annexin V stainings were performed (Figure 2(b)). For all tested conditions, the percentage of annexin V-positive cells remained below 5%, demonstrating that neither monocytes nor T-cells are in a more apoptotic state at the beginning of the culture experiments after prolonged sample aging. Next, the effect of sample aging on cell functionality was investigated by the measurement of the mitochondrial membrane potential (MMP), a marker for cellular stress (Figure 2(c)). Also based on MMP, no effect of sample aging was detected that could explain the significant reductions in LPS-induced cytokine responses. Finally, it was investigated whether the impaired LPS responses in aged blood samples could be explained by a reduced recognition of the trigger. However, sample aging did not significantly affect TLR4 expression on monocytes (Figure 2(d)).

### 3.3. Sample Aging Does Not Affect the Number of Responding Cells

**Figure 3:**
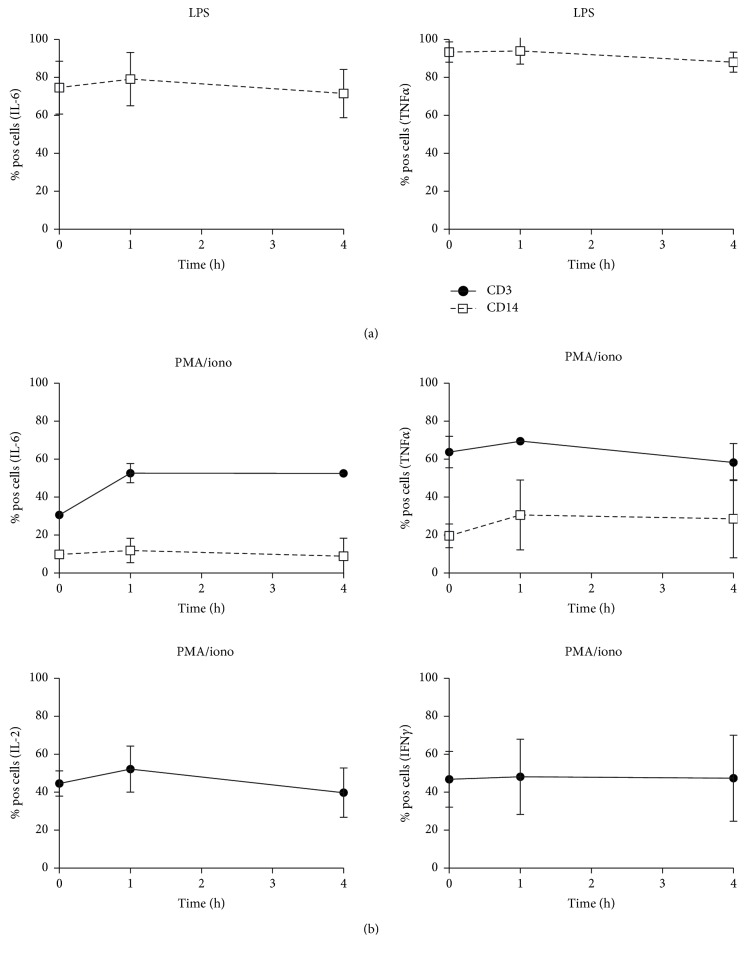
Cytokine-producing cells in whole blood cultures (average plus SD). The whole blood of 5 donors was stimulated for 3 hours with LPS (a) or PMA/ionomycin (b) in the presence of brefeldin A to block cytokine secretion. The percentage of positive cells for intracellular cytokines was quantified for T-cells (continuous lines) and monocytes (dashed lines). The *x*-axis indicates the sample age (time span between blood collection and start incubation). The blood was stored at room temperature. T-cells and monocytes remained negative for IFN*γ* staining after LPS stimulation.

To explore whether the impaired LPS response after sample aging could be explained by a reduction in responding number of cells, additional experiments were conducted, but now with intracellular cytokine production as the endpoint. Whole blood cultures were started directly after blood collection and with a delay of 1 and 4 hours and storage at room temperature. After LPS stimulation, the percentage of cytokine-producing monocytes was high: approximately 80% produced IL-6, and 95% produced TNF*α* (Figure 3(a)). T-cells and monocytes remained negative for IFN*γ* staining after LPS stimulation (data not shown). In response to PMA/ionomycin, approximately 45% of the T-cells produced IL-2 and IFN*γ*, 30% produced IL-6, and 65% produced TNF*α* (Figure 3(b)). Monocytes responded to PMA/ionomycin as well: approximately 10% produced IL-6, and 20% produced TNF*α*. Importantly, sample aging did not affect the number of monocytes or T-cells responding to LPS or PMA/ionomycin (Figures 3(a) and 3(b)).

### 3.4. The Addition of Culture Medium Does Not Preserve the Whole Blood Response

**Figure 4:**
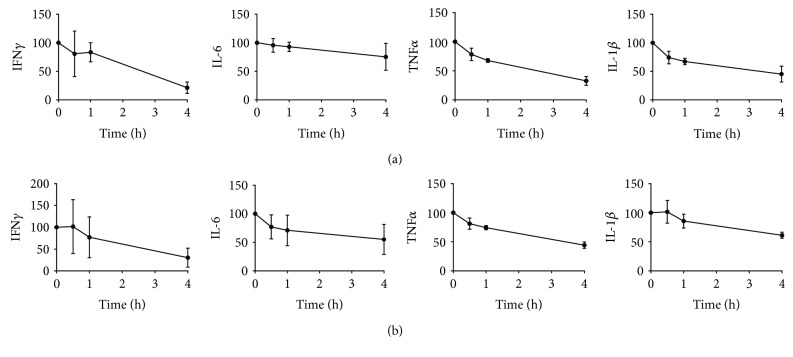
Cytokine release in whole blood culture supernatants; RPMI effect (as % of the response at *t* = 0, average plus SD). The whole blood of 3 donors was stimulated for 24 hours with LPS in the absence of RPMI (a) or diluted 1 : 1 with RPMI during aging (b). The *x*-axis indicates the sample age (time span between blood collection and start incubation). The blood was stored at room temperature.

**Figure 5:**
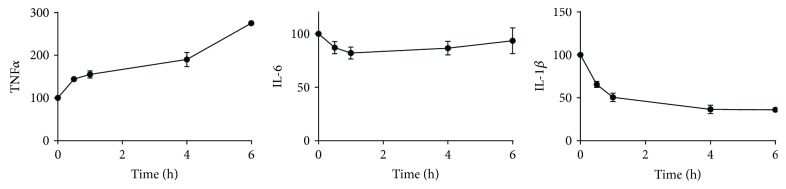
Cytokine release in PBMC culture supernatants (as % of the response at *t* = 0, average plus SD). Isolated PBMCs of 2 donors were stimulated for 24 hours with 2 ng/mL LPS directly after isolation or after aging up to 6 hours in RPMI + 10% FBS. The *x*-axis indicates the sample age (time span between PBMC isolation and start incubation). Cells were stored at room temperature. For IFN*γ*, most samples remained below LLOQ.

To assess whether the addition of culture medium prevents the sample aging-dependent loss of cellular responsiveness to LPS, RPMI1640 was added to the whole blood samples directly after blood collection (Figure 4). The addition of RPMI to the blood cultures did not result in the preservation of cell responsiveness: LPS-induced cytokine responses were affected to the same extent by sample aging in the absence and presence of RPMI. To test whether the sample aging effect could relate to influences of dying granulocytes or granulocyte products, an experiment was performed with PBMCs. Cells were stimulated with 2 ng/mL LPS directly after PBMC isolation (*t* = 0 h) or after up to 6 hours of aging (Figure 5). The sample aging effect on LPS responsiveness was comparable between the PBMC experiment (Figure 5) and earlier whole blood experiments (Figures 1 and 4) concerning IL-6 (mild reduction in cytokine release) and IL-1*β* (significant reduction in cytokine release). Interestingly, the effect on LPS-induced TNF*α* release was opposite between both settings: whereas sample aging reduced TNF*α* release in the whole blood (Figures 1 and 4), it strongly enhanced TNF*α* release in PBMCs (Figure 5).

## 4. Discussion and Conclusions

Human whole blood cultures are widely used for the investigation of physiological pathways and drug effects *in vitro*. Detailed information on the effect of “sample aging” (the time span between blood collection and experimental start) on the experimental outcome is not readily available in the public domain. This is an important knowledge gap, especially since whole blood-based pharmacodynamic assays have become increasingly important for the guidance of first-in-human clinical pharmacology studies with investigational medicinal products. Whole blood challenges are commonly applied to study the effect of immunomodulatory compounds on innate immune responses, such as ex vivo LPS and PHA stimulations [17–19]. No strict protocols or criteria apply for such whole blood cultures, and the main factor that potentially confounds experimental outcome is the “sample age,” the time span between blood collection and the start of the experiment. To provide more insight into the effect of sample aging on innate immune responses induced ex vivo, we stimulated whole blood samples with cell-specific immune triggers (LPS, PMA/ionomycin, and SEB). We studied the effect of sample aging on the ability of immune cells to respond to these triggers. The whole blood was minimally diluted (9% dilution) to resemble *in vivo* conditions as close as possible.

Sample aging at room temperature, but also at 4°C or 37°C, profoundly inhibited LPS-induced cytokine release. At room temperature, the reduction ranged from 20–50% after 30 minutes to 80–100% after 10 hours, with the strongest reductions observed for IFN*γ* and the smallest reduction for IL-6. An LPS-driven cytokine response in whole blood samples is mainly monocyte-derived [20]. PMA/ionomycin- and SEB-induced cytokine releases, both mainly T-cell-driven, were also reduced by sample aging but to a lesser extent (20–50% after 24 hours). Apparently, in a whole blood setting, monocytes are more affected by sample aging than T-cells. PMA/ionomycin stimulation of the whole blood drives a T-cell response, but also directly activates monocytes. For example, monocytes produce IL-2 upon PMA/ionomycin stimulation [21]. We observed IL-6 and TNF*α* production by monocytes after PMA/ionomycin stimulation. Therefore, the slight reduction in IL-6 and TNF*α* in aged whole blood samples stimulated with PMA/ionomycin is probably explained by a lower responsiveness of the monocyte fraction, and not of the T-cell fraction.

Previous reports on the effect of sample aging show that monocyte responses do not diminish after prolonged sample storage [13, 14]. Schultz et al. concluded that prolonged sample storage resulted in a decreased lymphocyte response, without affecting monocyte responsiveness [14]. However, these conclusions were based on intracellular cytokine staining only. In our experiments, intracellular cytokine staining also showed that the number of LPS-responding cells was not impacted by sample aging, so the reduced responsiveness to LPS could not be explained by fewer cells responding to the trigger. In previous experiments, whole blood cultures were diluted with culture medium providing nutrients for the cells [14, 22], which may preserve cell responsiveness during prolonged periods of sample storage. Therefore, we repeated our experiment with RPMI1640 culture medium added to the aging samples, but this did not prevent or reduce sample aging-dependent decreases in cytokine production.

Since monocytes can die via an apoptotic process in the absence of specific activation stimuli [23, 24], we investigated whether reduced LPS responsiveness could be explained by cell death or cellular stress. However, we did not find any indication for a sample aging-dependent reduction in the number of viable immune cells, elevated apoptotic cells, or altered mitochondrial membrane potential. This is in line with literature reporting that apoptosis in whole blood cultures only develops after at least 24 hours of sample storage [3]. Since the uptake of apoptotic cell fragments may suppress the cytokine production by macrophages [25], the conclusion that reduced cytokine responses in our experiments do not coincide with cell death or cellular stress which is informative and suggests that other physiological mechanisms may explain the observed effects of sample aging. We also demonstrated that reduced cytokine release in aging samples did not correlate with downregulated TLR4 expression or with an enhanced IL-10 production (data not shown). Thus, we speculate that sample aging induces an inhibitory pathway downstream from TLR4 in monocytes. Alternatively, changes in the expression of cytokine receptors on monocytes may have caused autocrine consumption of cytokines, but this was not investigated.

All the cytokine release experiments described in this manuscript were performed with incubation durations of 24 hours. This time span is sufficiently long to allow not only primary LPS-driven responses but also secondary leukocyte responses initiated by LPS-induced factors or even by factors induced by sample aging. One potentially relevant factor driving secondary responses is granulocyte-related products. For example, granulocyte contamination in PBMC cultures reduced T-cell responses to PHA and fMLP [26]. Granulocytes are known to have a short lifespan and to be sensitive to sample handling. Spontaneous activation of granulocytes occurs after 6–8 hours after venipuncture [26]. In our experiments, no apoptosis of granulocytes was observed in the first 10 hours after sample collection (data not shown). Since we did not assess the level of granulocyte activation, it is theoretically possible that this may have had an effect on the immune responses in our whole blood cultures. However, we showed that sample aging-dependent alterations in some LPS-induced cytokine responses were not only observed in the whole blood but also in PBMC cultures, suggesting that granulocyte-derived factors alone do not explain the observed effects of sample aging.

Significant levels of IFN*γ* were released upon LPS, SEB, and PMA/ionomycin stimulation in whole blood cultures. However, intracellular staining showed that LPS stimulation did not induce IFN*γ* production in either monocytes or T-cells. There are different potential explanations for this observation. Possibly, a different cell type accounted for the IFN*γ* production after LPS stimulation, for example, NK cells and B cells, are known to produce IFN*γ* upon induction of innate immune responses [27]. Alternatively, LPS-induced IFN*γ* release may have been a secondary to a primary LPS-driven effect, and the incubation time for the intracellular cytokine experiments was too short to allow such a secondary response. Further investigation into this observation falls beyond the scope of this paper.

These results underline the importance of the use of fresh samples when investigating innate immune responses in the whole blood. Given the ever increasing application of whole blood challenge tests as pharmacodynamic readout measure in early phase clinical pharmacology trials, a better understanding of the conditions affecting the outcome of such tests is critical. We demonstrated that sample aging primarily affects monocyte responses and that this cannot be explained at the level of cell viability or ligand recognition.

## Data Availability

The data used to support the findings of this study are available from the corresponding author upon request.
